# The Meaning of Health in the Era of Value-based Care

**DOI:** 10.7759/cureus.1042

**Published:** 2017-02-21

**Authors:** Joseph E. Balog

**Affiliations:** 1 Public Health and Health Education, The College at Brockport, SUNY

**Keywords:** ethics, concept of health, meaning of health

## Abstract

In an era of value-based care, the practice of medicine and other health professions have been drawn to subjective, comprehensive and multidimensional views of health such as the World Health Organization(WHO) concept that defines health as a state of complete physical, mental, and social well-being and not merely the absence of disease or infirmity. This paper, through a philosophical analysis, demonstrates that health is not multidimensional and is a natural phenomenon. A philosophical discussion contends that health must realistically and logically reside in the person and this requires it to be a physical state. This paper also illustrates that, in the popular language of health, many times, health professionals: (1) inappropriately view health as a subjective human construct as opposed to viewing health as an objective phenomena, (2) confuse what is desired and valued as a good life with what is good health, and (3) fail to recognize the vital distinction between what affects health and what is health. A meaning of health is offered through several examples and arguments that demonstrate why health is a state of physical well-being or physical fitness that is defined by how well the body is functioning in accordance with its natural design and how well this natural design affords individuals the ability to achieve essential functional objectives of humans on the biological and person level.

## Introduction and background

There is something interesting, peculiar, yet troubling about the challenge of defining health in the era of value-based care. Attending this challenge involves the task of considering an idea that health may be a subjective, human construct that is relative to time, place and person, to borrow a concept from epidemiology.  This is quite different than having health professionals view health as an objective and natural phenomena that reside in humans and has universal qualities that are not influenced by what era one lives in or by what multifarious values one holds.

A popular concept of health and a concept that is reflected in the overwhelming practice of health care is the notion that health is the opposite or absence of disease. An underlying assumption of this thinking encompasses the notion that people in their natural and normal state are healthy and individuals with disease, illness or injury are looked upon as being unhealthy. Throughout history, alternative views have been offered to replace this idea of health and perhaps none more famous than the World Health Organization's (WHO) views states health is a state of complete physical, mental, and social well-being and not merely the absence of disease of infirmity [1]. However, this paper argues that the WHO concepts of health and other relational views are peculiar and troubling. Health seen as the absence of disease is perhaps not the most attractive or aspirational view in the health fields. However, in the practice of health care, with its emphasis on reducing harm in the forms of morbidity and mortality rates for the purpose of protecting, maintaining and promoting the natural state of normal physical functioning health offers an appropriate and reasonable idea of health. 

Understanding what is health is a difficult task. One very important reason for this difficulty revolves around the idea that the concept of health calls into question the fundamental challenge of determining whether health, in the language of ethics, is a normative or non-normative concept. In other words, is health an idea that humans create related to cultural values and social norms or is it a natural phenomenon that is discovered and described in congruence with an objective, factual and natural design. This paper argues that health is in line with the latter perspective.

## Review

Unwittingly or not, when health professionals entertain or accept the idea that health is a subjective and relativistic concept, this position opens up the door for value judgments and social ideologies to delineate the meaning of health. This is a peculiar and troubling practice because such views often blur the line between what we desire as a good life with what is good health. For example, in a textbook on health and wellness, the authors stated that people with disease may live joyful, positive, healthy lives [2]. Yes, it is possible that a person who has diabetes mellitus can experience joy in his or her life and be a very happy and productive person, but it is not true that this individual is healthy. A disease that interferes with the normal physical functioning of the body and which can be life threatening is not an example of health. It’s an example of unhealthiness.

Complications related to diabetes can include heart attacks, strokes, blindness, kidney failure, lower limb amputations and even death. In the US, over 29.1 million people have diabetes and in 2014, over 76,000 people succumbed and died from this disease [3-4]. Worldwide, it is estimated that over 366 million people have diabetes and by 2030, the prevalence will rise to 552 million [5]. The prevalence and resultant harm from diabetes should confirm that health professionals are correct in seeing diabetes as a disease and individuals who have this disease are, in fact, unhealthy, even if varying degrees of dysfunction, health and harm exist.

To continue the example of diabetes, let’s explore another way of how a subjective desire for a good life slips into the language of health. The desire to value all individuals, regardless of whether they have diabetes or not, is notable. It is a good thing to value people’s lives. It is also a good thing to want people to have good lives. It is also reasonable to think that good health is a part of a good life. However, whether people are good or not, or whether they live good lives or not, are traits and qualities that are independent of having good health. Living a good life may be desirable and a good life may affect health, but the presence of disease, illness or injury, and their reality to do harm, is separate and independent realities in life. Good people and scoundrels can both become diabetics; this is not unusual. In either case, if we value health, people, and life, then disease such as diabetes is undesirable for it threatens people's health and their lives. It is not the other way around.Health does not threaten a good life.

Not clearly distinguishing between what affects health and what is health, also contributes to the confusion and misunderstanding of the meaning of health. In epidemiological terms, the agent, for example, a microorganism such as a poliovirus (a human enterovirus of the Picornaviridae family) can exist in the environment that cause paralytic poliomyelitis in an individual. The agent itself is not unhealthy nor is the environment unhealthy. Rather, humans make judgments and diagnoses about the presence of health and disease by looking at whether or not the agent in the environment infected the host and caused deleterious effects in the host, the individual. To determine whether or not the individual is in a disease state, ill or injured (states of unhealthiness), health professionals screen and evaluate the physical condition and physiological functioning of the individual. Next, health experts compare their findings to an established natural design and to an established standard for normal bodily functioning of humans. If deviance from a natural design of physiological functioning is found to disrupt normal bodily activity, then a person will be considered being in an unhealthy state and needing health care.

Continuing with the example of polio, if the poliovirus infection was asymptomatic and an initial diagnosis did not recognize the presence of disease then it is the judgment made by the health expert that is incorrect and not the fact that the virus exists and has the potential to cause pathological dysfunction and harm. Now, suppose that the viral replication continues and the virus is no longer limited to the alimentary tract. In about 24% of infected individuals, and after an incubation period of about seven to ten days, an infected individual will likely develop clinical signs of disease such as fever, headache and sore throat [6]. These are all signs of abnormal bodily functioning that represent a physiological deviance from normal and appropriate bodily functioning. In these cases of polio, there are by definition, underlying pathological findings to explain this abnormal condition. Thus, even though an observer has not examined the internal central nervous system to see if abnormality is present, the health expert would have, based on past pathophysiological criteria, diagnosed the signs and symptoms mentioned above as deviant and abnormal. By using factual and universal pathophysiological criteria, the infected person is ill and unhealthy.

In an estimated one percent of poliovirus infections, paralytic poliomyelitis can occur when the virus enters the central nervous system and destroys nerve cells that activate skeletal muscles [6]. In this situation, it is possible for the infected person’s muscles to lose their normal function caused by a lack of nervous enervation that can evolve into acute flaccid paralysis. Is this person healthy? No, this person is not healthy. Paralytic poliomyelitis clearly represents the malfunctioning of the body and it expresses the antithesis of a universal and evolutionary design of human functioning. Individuals can adapt to living with polio and live productive or nonproductive lives. Nonetheless, no matter how successful or unsuccessful these individuals are in facing the challenge of polio, it is still unreasonable to claim that at the point of infection and paralysis health experts can’t make a decision about health by using universal and normal standards of physiological functioning. The idea that evaluators of health should wait and see whether individuals grows up and becomes psychologically and sociologically well-adjusted to their condition in life appears quite peculiar.

Noncommunicable diseases follow the same model. A lack of exercise, smoking cigarettes, the heavy drinking of alcohol, or being involved in a stressful relationship may not be good for a person’s health, but these factors are not health; they are factors or behaviors that can have an effect on health. A lack of exercise is not healthy or unhealthy. A lack of exercise is a lack of exercise. A person who exercises may have improved cardiovascular fitness, and this state of fitness may be the outcome of participating in regular regimes of exercising. However, the person’s health is determined by an evaluation of the physical presence of statistical, clinical, or normative criteria such as resting heart rate or blood pressure.

Relational and subjective views of health, such as the WHO’s concept of health, expand the description of health from a physical domain and into a “multidimensional” view of the human condition. Multidimensional views are attractive, but they should also attract analytical challenges to their reasonableness. These multidimensional views represent the thinking that health and humans are more than physical beings. A questionable assumption made in such views is that humans are more than the sum of their physical being. However, the above examples of polio demonstrate how these views break down when disease, illness, and injury present and cause interference with abnormal physiological functioning and bodily harm.

If one believes that health is multidimensional and includes criteria such as psychological, social and spiritual health, then this notion begs the question: “Where does health reside?”. Consider the idea of social health, if there is such a thing, then it must reside somewhere outside of individuals and exist among individuals. For example, suppose in a small worksite office there are ten people who work together but they all don’t get along. The boss of this small company is very demanding and three employees constantly complain about the workload that is assigned to them. The three employees feel that they were unfairly treated because they believe they were given a greater amount of work than their co-workers. They feel stressed and seek medical advice to help them manage their stress. The other six employees believe they have been given similar workloads and appear to be fine with their assignments.

There does appear to be problems of interpersonal relationships in this office, but are these unhealthy relationships, and/or are these people socially unhealthy?. If we are to take this scenario seriously as an issue of health, then an evaluator of health is going to have to look at health existing somewhere “outside” of individuals and existing somewhere in a “space” where these employees co-exist together. The evaluator of social health is going to have to make judgments about the quality of interactions among these individuals. This example of social health demonstrates how the idea of social health is ludicrous. Health does not reside outside of a person and it shouldn’t be determined by some subjective thoughts about how should people get along with each other at work or at any other place. Interpersonal relationships are simply issues of interpersonal relationships and not an issue of health. Health resides in people, in their bodies, and not in the dialogues or in the quality of interpersonal behaviors among people. Individuals in our lives and the quality of relationships in our lives can affect our health. These relationships can be good, bad, joyful, irritating, stressful, pleasurable, and so on. Social interactions can stimulate physiological reactions in our bodies, but they are not health, nor do they contain health; health physically resides in an individual’s body.

Sociological models and public health models have offered valuable information about the idea that health is communal and the health of individuals is not detachable from the social context and conditions of their environment. This reality has always been true and in modern times epidemiological studies document the social, environmental and economic conditions and their effect on health. The epidemiological evidence in these works established the groundwork for views on health disparities, inequalities in health, social determinant of health and the social gradient in health. For example, MacDorman and Mathews report that disparities in infant mortality rates in the U.S. are strongly related to racial, ethnic and economic factors, and these disparities have been apparent since these data began to be collected more than 100 years ago [7]. Noncommunicable diseases follow similar trends. For example, the Centers for Disease Control and Prevention (CDC) reported that the marked racial/ethnic and socioeconomic factors are related to disparities in diabetes. For example, the prevalence of diabetes is greater in socioeconomically disadvantaged populations than in advantaged socioeconomic populations [8].

Medicine needs to be concerned with determinants of health that have traditionally fallen outside of a clinical and medical context and more into a public health context. Medicine and the health professions also need to be concerned about issues of access to health care, the relationship between income disparities and health inequities, and other social and environmental issues and factors related to securing health. And, medicine and the health professions should strategize about what role they should play in attempting to modify the social context of people’s lives. However, professional decisions that lead to the appropriate social and moral obligations and actions of medical and health-related professions will not change the meaning of health.

Simply put, health must reside in the person and this requires it to be a physical state. If this is not the case, then in an era of valued-based care, health professionals may think of health as no more than a subjective and relative concept that is invented and defined in relationship to cultural values and social norms. Such views, as warned about above, could lead to peculiar ideas such as social health or views that don’t adequately distinguish between what humans desire as a good life and what human desire as good health, and what affects health as opposed to what is health. If health exists, then it must be a part of what it means to be human and to live within the human condition. Health is a state of physical well-being or physical fitness and it is defined by how well the body is functioning in accordance with its natural design and how well this natural design affords individuals the ability to achieve essential functional objectives of humans on a biological and person level.

The subject of mental health is a complex issue and requires a comprehensive discussion about its etiology and understanding of what is its true meaning. However, to briefly introduce and attend to this topic and how it is related to the task at hand, consider the idea that mental health is the maintenance of self-consciousness, the ability to make assessments of our thoughts, feelings and actions and the potential to make rational judgments. Health, in this sense, is the possession of a central nervous system that is physically functioning in accordance with its natural design. In other words, being physically fit enough, as a Surgeon General’s Report on mental health implied, to have the mental functions of thinking, reasoning, feeling, and thoughts about purposive behavior [9].

The field of neuroscience offers supporting evidence for this view and has suggested that the mind and body are inseparable. Within the “mind,” the brain contains the physiological and mental functions of thinking, mood, and purposive behavior; it is an expression of biological processes that are physical activities derived from the brain. Thus, mental health, in this sense, is a physical state of physiological and mental functions. Judgments about the quality or value of these mental functions are not health nor are the opinions of others about the intended or actualized behaviors related to thoughts, health. Health is proper functioning of the central nervous system that affords individuals levels of mental functioning that has the ability to achieve the essential functional objectives of humans on a biological and human level. The ability to possess self-conscious, rational and quality thoughts may lead to productive and fulfilling lives. However, these state of affairs are outcomes of mental functioning, not the physiological functioning itself. In our culture, the resulting thoughts, feelings, and behaviors of physiological and mental functioning are viewed and defined as mental health, but it would be more accurate to define these results as expressions of physiological and mental functioning. 

## Conclusions

Four basic conclusions may be drawn from the above discussion. The first is that health resides within individuals, it is natural phenomena, it is a state of physical well-being or physical fitness, and it is defined by how well the body is functioning in accordance with its natural design and how well this natural design affords individuals the ability to achieve essential functional objectives of humans on a biological and person level.

Second, in medicine, and in the health care professions in general, the above physiological rooted view of health is perhaps not as attractive as subjective and relational views such as the WHO concept of health. However, such human constructs that offer more comprehensive and/or multidimensional views of health tend to confuse what is health with what is desirable as a good life.

Third, health professionals understand how health is communal and the health of individuals is not detachable from the social context and environmental conditions in their lives. They see the importance of attending to the physical, social, environmental and economic conditions that affect health, but they should also understand the distinction between what affects health and what is health.

Finally, practicing medicine in an era of value-based care does not mean that medicine is not interested in problems of virtue and life. Valuing goodness and life is at the heart of medicine. However, this paper does caution medicine in an era of value-based care not to have its practice compromised by subjective and attractive views of health. Relational views of health distract the practice of medicine from its vital function of protecting and maintaining the physical health of individuals. This view of health does not reduce the value of medicine, nor does it neglect the importance of dealing with the “whole” person; rather, it solidifies the need for medicine to decrease morbidity and mortality rates so humans can increase their opportunity to live a good and healthy life. This value for medicine transcends time and place and it should not be influenced by what era one lives in or what multifarious values cultures may hold.
